# Strategies to implement evidence-informed decision making at the organizational level: a rapid systematic review

**DOI:** 10.1186/s12913-024-10841-3

**Published:** 2024-04-01

**Authors:** Emily C. Clark, Trish Burnett, Rebecca Blair, Robyn L. Traynor, Leah Hagerman, Maureen Dobbins

**Affiliations:** 1 National Collaborating Centre for Methods and Tools, McMaster University, McMaster Innovation Park, 175 Longwood Rd S, Suite 210a, Hamilton, ON L8P 0A1 Canada; 2 School of Nursing, McMaster University, Health Sciences Centre, 2J20, 1280 Main St W, Hamilton, ON L8S 4K1 Canada

**Keywords:** Evidence-informed decision making, Evidence-based practice, Knowledge translation, Knowledge mobilization, Implementation, Organizational change

## Abstract

**Background:**

Achievement of evidence-informed decision making (EIDM) requires the integration of evidence into all practice decisions by identifying and synthesizing evidence, then developing and executing plans to implement and evaluate changes to practice. This rapid systematic review synthesizes evidence for strategies for the implementation of EIDM across organizations, mapping facilitators and barriers to the COM-B (capability, opportunity, motivation, behaviour) model for behaviour change. The review was conducted to support leadership at organizations delivering public health services (health promotion, communicable disease prevention) to drive change toward evidence-informed public health.

**Methods:**

A systematic search was conducted in multiple databases and by reviewing publications of key authors. Articles that describe interventions to drive EIDM within teams, departments, or organizations were eligible for inclusion. For each included article, quality was assessed, and details of the intervention, setting, outcomes, facilitators and barriers were extracted. A convergent integrated approach was undertaken to analyze both quantitative and qualitative findings.

**Results:**

Thirty-seven articles are included. Studies were conducted in primary care, public health, social services, and occupational health settings. Strategies to implement EIDM included the establishment of Knowledge Broker-type roles, building the EIDM capacity of staff, and research or academic partnerships. Facilitators and barriers align with the COM-B model for behaviour change. Facilitators for capability include the development of staff knowledge and skill, establishing specialized roles, and knowledge sharing across the organization, though staff turnover and subsequent knowledge loss was a barrier to capability. For opportunity, facilitators include the development of processes or mechanisms to support new practices, forums for learning and skill development, and protected time, and barriers include competing priorities. Facilitators identified for motivation include supportive organizational culture, expectations for new practices to occur, recognition and positive reinforcement, and strong leadership support. Barriers include negative attitudes toward new practices, and lack of understanding and support from management.

**Conclusion:**

This review provides a comprehensive analysis of facilitators and barriers for the implementation of EIDM in organizations for public health, mapped to the COM-B model for behaviour change. The existing literature for strategies to support EIDM in public health illustrates several facilitators and barriers linked to realizing EIDM. Knowledge of these factors will help senior leadership develop and implement EIDM strategies tailored to their organization, leading to increased likelihood of implementation success.

**Review registration:**

PROSPERO CRD42022318994.

**Supplementary Information:**

The online version contains supplementary material available at 10.1186/s12913-024-10841-3.

## Background

There exist expectations that decisions and programs that affect public and population health are informed by the best available evidence from research, local context, and political will [1–3]. To achieve evidence-informed public health, it is important that public health organizations engage in and support evidence-informed decision making (EIDM). For this review, “public health organizations” refers to organizations that implement public health programs, including health promotion, injury and disease prevention, population health monitoring, emergency preparedness and response, and other critical functions [4]. EIDM, at an organizational level, involves the integration of evidence into all practice decisions by identifying and synthesizing evidence, then developing and executing plans to implement and evaluate changes to practice [2, 5, 6]. EIDM considers research evidence along with other factors such as context, resources, experience, and patient/community input to influence decision making and program implementation [2, 3, 7, 8]. When implemented, EIDM results in efficient use of scarce resources, encourages stakeholder involvement resulting in more effective programs and decisions, improves transparency and accountability of organizations, improves health outcomes, and reduces harm [3, 7, 8]. Therefore, it is important that EIDM is integrated into organizations serving public health.

Driving organizational change for EIDM is challenging due to the need for multifaceted interventions [9].While there are systematic reviews of the implementation of specific evidence-informed initiatives, reviews of implementation of organization-wide EIDM are lacking. For example, Mathieson et al. and Li et al. examined the barriers and facilitators to the implementation of evidence-informed interventions in community nursing and Paci et al. examined barriers in physiotherapy [10–12]. Li et al. found that implementation of evidence-informed practices is associated with an organizational culture for EIDM where staff at all levels value and contribute to EIDM [12]. Similarly, Mathieson et al. and Paci et al. found that organizational context plays an important role in evidence-informed practice implementation along with organizational support and resources [10, 11]. While these reviews identify organizational context, culture and support as crucial for the implementation of a particular evidence-informed practice, they do not identify and describe sufficiently what and how an organization evolves to consistently be evidence-informed for all decisions and programs and services it delivers.

Primary studies have explored how building capacity for staff to find, interpret and synthesize evidence to develop practice and program recommendations may contribute to EIDM [13–16]. In 2019, Saunders et al. completed an overview of systematic reviews on primary health care professionals’ EIDM competencies and found that implementation of EIDM across studies was low [9]. Participants reported insufficient knowledge and skills to implement EIDM in daily practice despite positive EIDM beliefs and attitudes [9]. In 2014, Sadeghi-Bazargani et al. and in 2018, Barzkar et al. also explored the implementation of EIDM and found similar results, listing inadequate skills and lack of knowledge amongst the most common barriers to EIDM [17, 18].

An underlying current in research for organizational EIDM is a focus on organizational change [13, 14, 19, 20]. To achieve EIDM across an organization, significant organizational change is usually necessary, resulting in substantial impact on the entire organization, as well as for individuals working there. However, while there are reviews of individual capacity for EIDM, there is minimal synthesized evidence describing EIDM capacity at the organizational level. This review seeks to address this research gap by identifying, appraising, and synthesizing research evidence from studies seeking to understand the process of embedding EIDM across an organization, with a focus on public health organizations.

The COM-B model for behaviour change was used as a guide for contextualizing the findings across studies. By integrating causal components of behaviour change, the COM-B model supports the development of interventions that can sustain behaviour change in the long-term. While there are numerous models available to support implementation and organizational change, the COM-B model was chosen, in part, for its simple visual representation of concepts, as well as its contributions to the sustainability of behaviours [21]. This model is designed to guide organizational change initiatives and distill complex systems that influence behaviour into simpler, visual representations. Specifically, this model looks at capability (C), opportunity (O) and motivation (M) as three key influencers of behaviour (B). The capability section of the COM-B model reflects whether the intended audience possess the knowledge and skills for a new behaviour. Opportunity reflects whether there is opportunity for new behaviour to occur, while motivation reflects whether there is sufficient motivation for a new behaviour to occur. All three components interact to create behaviour and behaviours can, in turn, alter capability, motivation and opportunity [21]. Selection of the COM-B model was also driven by authors’ extensive experience supporting public health organizations in implementing EIDM, which observed enablers for EIDM that align well with the COM-B model, such as team-wide capacity-building for EIDM, integration of EIDM into processes, and support from senior leadership [20, 22, 23]. The COM-B model has been used to map findings from systematic reviews examining the barriers and facilitators of various health interventions including nicotine replacement, chlamydia testing and lifestyle management of polycystic ovary syndrome [24–26]. This review has a broader focus and maps barriers and facilitators for organization-wide EIDM to the COM-B model.

Overall, EIDM is expected to be a foundation at public health organizations to achieve optimal health of populations. However, the capacity of public health organizations to realize EIDM varies considerably from organization to organization [14, 22, 27–29]. This rapid review aims to examine the implementation of EIDM at the organizational level to inform change efforts at Canadian public health organizations. The findings of this review can be applied more broadly and will support public health organizations beyond Canada to implement change efforts to practice in an evidence-informed way.

## Methods

### Study design

The review protocol was registered with the International Prospective Register of Systematic Reviews (PROSPERO; Registration CRD42022318994). The review was conducted and reported following the Preferred Reporting Items for Systematic Reviews and Meta-Analyses (PRISMA) statement for reporting systematic reviews and meta-analyses [30]. A rapid review approach was used, since the review was requested to be completed by the National Collaborating Centre for Methods and Tools’ Rapid Evidence Service within a specific timeline, in order to inform an organizational change initiative at a provincial public health organization in Canada [31]. Given the nature of the research question, a mixed methods rapid systematic review approach was taken, with guidance from the Joanna Briggs Institute (JBI) Manual for Evidence Synthesis [32].

### Information sources and search strategy

The search was conducted on March 18, 2022. The following databases were searched from 2012 onward: Medline, Embase, Emcare, Global Health Database, PsycINFO, Web of Science. Each database was searched using combinations and variations of the terms “implement*”, “knowledge broker*”, “transform*”, “organizational culture”, “change management”, “evidence-based”, “knowledge translation”, and “knowledge mobilization”. Additionally, publications by key contributors to the field were reviewed. The full search strategy is included in Appendix 1.

Studies were screened using DistillerSR software. Titles and abstracts of retrieved studies were screened by a single reviewer. Full texts of included studies were screened by a second reviewer and reviewed by a third. Screening was not completed in duplicate, consistent with a rapid review protocol [31]. To minimize the risk of bias, a subset of 100 retrieved articles were screened in duplicate at the title and abstract stage to ensure consistency across reviewers. Of this subset, there were four articles with conflicting decisions, which were discussed amongst screeners to clarify inclusion criteria.

### Eligibility criteria

English-language, published primary studies with experimental or observational designs were eligible for inclusion. Review papers, such as literature and systematic reviews, were excluded to ensure that details regarding implementation of initiatives were captured without re-interpretation or generalization by review authors. Grey literature was not included. Eligibility criteria are outlined below in terms of a PICO (Population, Intervention, Comparison, Outcome) structure [33].

#### Population

Studies conducted with public sector health-related service-delivery organizations were eligible for inclusion. This included public health departments and authorities, health care settings and social services. Studies focused on departments or teams within an organization, or on entire organizations, were also eligible for inclusion. Studies conducted in private sectors or academic institutions were excluded to narrow the focus of the review.

#### Intervention

Interventions designed and implemented to shift teams, departments, or organizations to EIDM in all decisions were eligible for inclusion. These can include initiatives where organizations establish roles or teams to drive organizational change for EIDM, or efforts to build and apply the knowledge and skill of staff for EIDM. These are distinct from implementation strategies for evidence-informed interventions. Eligible interventions were applied to a team, department, or organization to drive change toward evidence use in decision making at all levels of the organizations.

#### Comparator

Studies that included any comparator or no comparator were included, recognizing that literature was likely to include case reports.

#### Outcomes

Outcomes measured either quantitatively or qualitatively were considered. These included behaviour change, confidence and skills, patient-level data such as quality indicators, evidence of EIDM embedded in organizational and decision-making processes, changes in organizational culture, and changes to budget allocation. Studies that reported primarily on implementation fidelity were excluded, since studies of implementation fidelity focus on whether an intervention is delivered as intended, rather than drivers for organizational change.

#### Setting

Studies conducted in the 38 member countries of the Organization for Economic Co-operation and Development (OECD) were included in this review to best align with the Canadian context and to inform organizational change efforts in public health within Canada [34].

### Quality assessment

The methodological rigour of included studies was evaluated using the JBI suite of critical appraisal tools [35]. Ratings of low, moderate, or high quality were assigned based on the critical appraisal results. Quality assessment was completed by one reviewer and verified by a second. Conflicts were resolved through discussion or by consulting a third reviewer.

### Data extraction

Data extraction was completed by a single reviewer and reviewed by a second. Data on the study design, setting, sector (e.g., public health, primary care, etc.), participants, intervention (e.g., description of learning initiatives, implementation strategies, etc.), outcome measures, and findings were extracted. To minimize the risk of bias, a subset of three included articles underwent data extraction in duplicate to ensure consistency across reviewers. There was good agreement between duplicate extraction, with variations in the format of extracted data but consistency in content.

### Data analysis

Quantitative and qualitative data were synthesized simultaneously, using a convergent integrated approach [32]. Quantitative data underwent narrative synthesis, where findings that caused benefit were compared with those that caused harm or no effect [36]. Vote counting based on the direction of effect was used to determine whether most studies found a positive or negative effect [36]. For qualitative findings, studies were grouped according to common strategies. Within these common strategies, findings were reviewed for trends in reported facilitators and barriers. These trends were deductively mapped to the COM-B model for behaviour change [37].

Due to the heterogeneity in study outcomes, the Grading of Recommendations, Assessment, Development and Evaluations (GRADE) [38] approach was not used for this review. Overall certainty of evidence was determined based on the risk of bias of included study designs and study quality.

## Results

**Fig. 1:**
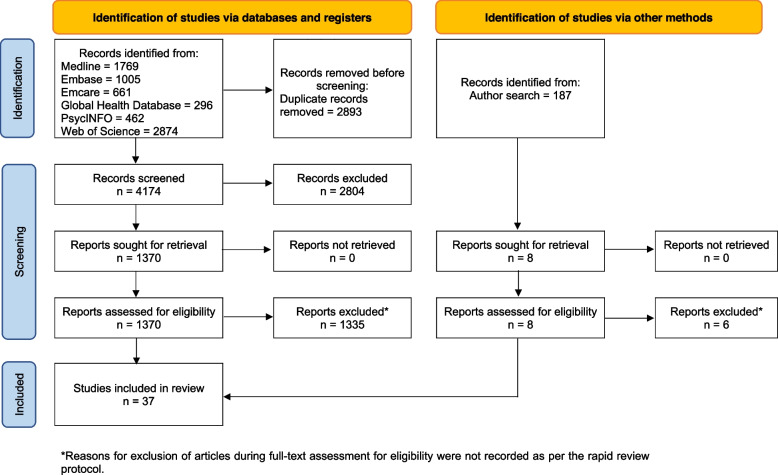
PRISMA 2020 flow chart

Database searching retrieved 7067 records. After removing duplicates, 4174 records were screened by title and abstract, resulting in 1370 reports for full text review. Of those 1370 records, 35 articles were included. Scanning the publication lists of key authors retrieved 187 records, of which eight were retrieved for full text review and two were included, for a total of 37 articles included in this review. See Fig. 1 for a PRISMA flow chart illustrating the article search and selection process.

### Study characteristics

The overall characteristics of included studies are summarized in Table 1. Of 37 included studies, most were conducted in primary care settings (*n* = 16) and public health settings (*n* = 16), with some in social services (*n* = 3), child and youth mental health (*n* = 1), and occupational health (*n* = 1). Most studies were conducted in the USA (*n* = 17), followed by Canada (*n* = 12), Australia (*n* = 5), and Europe (*n* = 3).

**Table 1:** Included studies of organization-wide implementation of EIDM

Reference	Study design, comparison	Setting, timeline	Sector	Participants	Intervention	Outcomes (Measurement tool)	Findings	Quality rating (Tool)
Studies of organization-wide implementation of EIDM								
Allen, 2018 [39]	Case report, no comparator	State health department, Georgia, USA, 2013–2016	Public health	Program staff across organization	Program staff received training for EIDM that included lectures, and small group problem-solving and discussion	Qualitative: EIDM facilitators and barriers (interviews)	Facilitators for EIDM: -Leadership support -Consistent internal messaging on EIDM -Close partnerships with evaluation teams -Requirement for evidence in proposals Barriers to EIDM: -Competing priorities -Limited budget for staff -Political conflicts in state and local agendas	High (Case report)
Allen, 2018 [40]	Qualitative	State health departments, USA, 2016	Public health	Leaders and program managers	State health departments to an intervention group that received EIDM training and support (See Brownson, 2017)	Qualitative: EIDM facilitators and barriers (structured interviews)	Facilitators for EIDM: -Leadership support -Developing structures and culture incorporating evidence based public health -Ongoing training -Building and maintaining partnerships with external partners Barriers to EIDM: -Funding/budget cuts -Lack of time -Lack of political will/support -Staff turnover	Moderate (Qualitative)
Augustino, 2020 [41]	Case report, no comparator	Military treatment facilities, USA, 2018	Primary care	Nursing staff at 4 facilities	An evidence-based practice facilitator role supported organization-wide EIDM teams through training, mentoring, and encouraging EIDM	Findings were described in a narrative case report	Facilitators for EIDM: -Incorporating the evidence-based practice facilitator into existing practice -Involving evidence-based practice facilitator in nursing meetings and committees -Aligning the evidence-based practice facilitator’s work with organizational priorities Barriers to EIDM: -Staff turnover -Lack of standardized evaluation of EIDM use	High (Case report)
Awan, 2015 [42]	Case report, no comparator	Centre for Addiction and Mental Health, Toronto, Ontario, Canada, 2013–2014	Primary care	Service providers, researchers at organization	An integrated care pathway, which relies on EIDM, was implemented for patients with concurrent major depressive disorder and alcohol dependence. Development of the integrated care pathway included evidence reviews, knowledge translation, process reengineering and change management	Quantitative: -patient symptom assessment and medication titration (Penn Alcohol Craving Scale, Quick Inventory for Depressive Symptoms scores and Beck Depression Inventory) Qualitative: -Facilitators and barriers (focus groups)	Evaluation of patient care found: -Lower program dropout (78% to 46% *p* < 0.05) -Reduction in depressive symptom severity (p-value not reported) -Reduction in heavy drinking days (42% to 23%, *p* < 0.04) Facilitators for EIDM: -Inclusion and frontline clinicians -Use of tools/templates (e.g., process maps, medication algorithms) -Team meetings Barriers to EIDM: -Lack of knowledge and skill for EIDM -Communication with referring providers	Moderate (Case report)
Bennett, 2016 [43]	Case report, no comparator	Large urban hospital, Australia, 18 months; dates not specified	Primary care	Occupational therapists in hospital	An EIDM capacity building program was implemented. The program included: -Educational outreach across organization -Teams working on clinical case studies -Allocating time for EIDM -Mentorship -Leadership support -Communication regarding EIDM -Development of EIDM processes and resources -Funding for an EIDM champion one day per week -Setting goals and targets for EIDM -EIDM reporting and evaluation	Qualitative: EIDM use, perceptions of organizational culture toward EIDM, EIDM facilitators and barriers (focus groups with clinicians and observations by the research team)	Facilitators for EIDM: -EIDM integration into roles -Buy-in to EIDM impact -Developing goals for EIDM -Access to mentors -Supportive leadership -Breaking down EIDM into manageable tasks -Journal club to discuss EIDM processes Challenges to EIDM: -Lack of EIDM knowledge and skill -Perceived lack of capability -Perceived lack of time and training -Competing priorities -Challenges with staff rotating between clinical teams	Moderate (Case report)
Breckenridge-Sproat, 2015 [44]	Single group pre-post study	Military hospitals, Washington, District of Columbia, USA, 18 months; dates not specified	Primary care	Nurses across hospitals	Unit-level mentors facilitated an educational mentoring program for EIDM. The intervention involved an organizational assessment, identification of facilitators and barriers, training EIDM mentors and EIDM implementation Librarian support, evidence-based practice education material, training modules were provided and supervised study team evidence-based practice projects were completed	Quantitative: -EIDM beliefs (Evidence-Based Practice Beliefs) -Organizational readiness and barriers to EIDM (Organizational Readiness for System-wide Integration of Evidence-Based Practice) -EIDM implementation (Evidence-Based Practice Implementation Scales)	Following the intervention, -Evidence based practice belief scores increased (*p* = 0.02) -Organizational readiness for EIDM scores increased (*p* < 0.01)	Moderate (Quasi-experimental study)
Brodowski, 2018 [45]	Case report, no comparator	Social service agencies, Kansas and Nebraska, USA, 2005–2011	Social work	Social service providing organizations	A workgroup of state-led agencies and federal partners developed a framework for infrastructure for EIDM, including federal policy for investing in evidence-based programs and quality improvement. Technical assistance was provided to community-based programs through a third party	Quantitative: Use of EIDM (annual reported funding for evidence-based programs) Qualitative: EIDM facilitators (interviews)	The percentage of funded programs that were evidence-based increased from 29 to 63% Facilitators for EIDM: -Strong infrastructure (outreach, training, fidelity assessment, supervision, management of the program -Availability of Technical Assistance: -Consideration of context when using EIDM to choose programs -Active engagement and collaboration with key stakeholders at all levels	High (Case report)
Brownson, 2017 [46]	RCT, control group	State health departments, USA, March 2014 and March 2015	Public health	Program staff across organization	State health departments randomized to: -Intervention group that received EIDM training workshop, and follow-up calls for technical assistance and supplemental activity planning and updates support -Control group that received links to electronic resources	Quantitative: perceived organizational skills and culture for EIDM (survey)	Following the intervention, -Perceived skills gaps decreased (*p* = 0.02) -Perceived supervisory expectation for use of EIDM increased (*p* = 0.006) -Use of evidence increased (*p* = 0.008)	Moderate (RCT)
Clark, 2022 [20]	Mixed methods, no comparator	Public health units, Ontario, Canada, 2015–2018	Public health	4–8 Staff members from each of 10 public health units	Senior leadership set organizational goals for EIDM during a facilitated focus group using the Is Research Working for you organizational assessment Knowledge translation specialist mentors delivered a Knowledge Broker mentoring program, including workshops, webinars, consultations and completion of a rapid review	Quantitative: -Attainment of organizational goals for EIDM (semi-structured interviews) Qualitative: -EIDM facilitators and barriers (semi-structured interviews)	Facilitators for EIDM: -Integration of EIDM into process through structures, processes, or templates -New or re-defined staff positions for EIDM -Leadership support -Culture of expectations of EIDM -Acceptance of time to learning and do EIDM Barriers to EIDM: -Lack of managers’ EIDM knowledge -Lack of protected time -Lack of staff buy-in -Lack of direction or plan for participants	High (Qualitative)
Dobbins, 2019 [47]	Single group pre-post study	3 Public health units, Ontario, Canada, 2010–2012	Public health	All staff at organization, senior leadership	Knowledge Brokers deployed to public health units supported individual capacity and organizational culture for EIDM. Knowledge brokers held workshops, mentoring, meetings with senior management and developed policies and processes for EIDM	Quantitative: -Knowledge, skills and behavioral assessment (survey) Qualitative: -EIDM facilitators and barriers (analysis of knowledge brokers journals)	Facilitators for EIDM: -Strong leadership support -Systematic integration of research evidence into decision-making processes -Access to librarian support -Committed financial and human resources -Staff interest and enthusiasm	Moderate (Quasi-experimental study)
Elliott, 2021 [48]	Case report, no comparator	Canadians Seeking Solutions and Innovations to Overcome Chronic Kidney Disease (Can-SOLVE CKD), Canada, dates not specified	Primary care	Clinicians, nurses	An integrated KT network (Can-SOLVE CKD) was established, including: -Central knowledge translation committee available for consultation -Support from external partners -KT planning templates -KT champions -KT virtual community of practice -KT online learning module	Findings were described in a narrative case report	Facilitators for EIDM: -Diverse knowledge base and members’ commitment to KT -Inclusion of patient’s perspectives Barriers to EIDM: -Generalizability to smaller project teams -Lack of KT skills among research and patient partners	Moderate (Case report)
Fernández, 2014 [49]	Case report, no comparator	The Cancer Prevention and Control Research Network, USA, dates not specified	Public health	National network	Workgroups across the network facilitated activities, including: -building the capacity of service providers for EIDM -developing technical assistance for KT -developing research partnerships -investigating implementation processes from other studies	Findings were described in a narrative case report	Successful EIDM activities were described, including the following. Network members translated and adapted the evidence-based Stanford Chronic Disease Self-Management program which was well attended and highly rated by participants. Cancer screening programs were adapted to the local context, increasing uptake among residents. Several partner universities have implemented workplace health promotion interventions	High (Case report)
Flaherty, 2021 [50]	Cluster RCT, control group	Outpatient child mental health clinics, New York, USA, dates not specified	Primary care	52 Child mental health care providers	4Rs and 2Ss Multiple Family Group intervention: -Providers received training and bimonthly supervision -Clinic Implementation Teams operated at agencies randomized to the intervention arm	Quantitative: Frequency of use of new techniques (Training Exposure and Utilization Scale), and organizational climate (Organizational Readiness for Change Scale)	Increased use of evidence-based interventions was associated with providers’ belief that organizational climate supported use of evidence-based interventions (b = − 0.33, SE = 0.11, *p* < 0.01)	Moderate (RCT)
Gallagher-Ford, 2014 [51]	Case report, no comparator	Large, complex healthcare system, USA, dates not specified	Primary care	Departments across an organization	A nurse administrator promoted and sustained a culture of evidence-based practice through the following activities: -Organizational assessments -Developing clinical nurse specialists as EIDM champions -Mentoring individuals through the change process	Findings were described in a narrative case report	Clinical nurse specialists have championed EIDM across the organizations. More than 13 projects for EIDM were initiated by clinical nurse specialists	Low (Case report)
Gifford, 2014 [52]	Qualitative	Large community healthcare organization delivering home and community healthcare, Ontario, Canada, 20-weeks; dates not specified	Public health	Management and clinical leaders from 4 units	Strategies to promote EIDM to nurse managers and clinical leaders in home healthcare were implemented, including, -Workshop on EIDM -Mentorship support from experienced “evidence facilitators” -Access to university library services -Information-sharing activities -Encouragement and recognition	Quantitative: EIDM use (Is Research Working for You? A Self-assessment Tool and Discussion Guide for Health Services Management and Policy Organizations) Qualitative: Usefulness of intervention, EIDM barriers and facilitators (semi structured interviews)	Following the intervention, participants reported: -More resources to conduct research -Staff contributions to EIDM discussions -More information about how evidence influenced decisions made in the organization (all *p* < 0.05) Facilitators for EIDM: -Ongoing education -Linking staff to EIDM experts -Social networking across organization -Recognition for EIDM work -Audit and feedback Barriers to EIDM: -Lack of time -Lack of knowledge, skills, and confidence -Conflicting priorities within the organization -Staff shortages	High (Qualitative)
Haynes, 2020 [53]	Case report, no comparator	Australian Prevention Partnership Centre, Australia, 5 years; dates not specified	Public health	Organization-wide, in partnership with research institutions	Six components for cross-sector collaborative partnerships for EIDM: 1. Partners involved at all stages 2. Communication efforts, e.g., forums, narrative reports 3. Skill development through workshops, webinars with experts 4. Cross-sector project teams 5. High-quality evidence syntheses 6. Ongoing surveys and opportunities for feedback	Quantitative: -Perceptions of leadership, governance, resource allocation, collaboration and engagement (Partnership survey) Qualitative: -Implementation and impact of projects (project evaluations) -Experiences and perceptions (semi-structured interviews)	Partners reported: -Translation of research into policy was built into processes -Many projects involved partners from different sectors -Communication across sectors and teams was adequate -Capacity building activities were valuable -Synergies were identified across projects	Moderate (Case report)
Hitch, 2019 [54]	Case report, no comparator	Public mental health service, major city in Australia, 2014–2016	Occupational therapy	Occupational therapists within the organization	Leadership role in KT established to support EIDM, complete research projects, build research capacity and culture, and create a database of research activity	Quantitative: -Attitudes towards EIDM (Evidence Based Practice Attitude Scale) -EIDM use (Evidence Based Practice Implementation Scale) -Staff perceptions of the Lead Research Occupational therapist role (survey)	After implementation of the KT role, -number of quality assurance and research activities increased (Cliffs Delta = 0.44; 95% CI = 0.22, 0.62) -no significant change in attitudes towards EIDM -staff viewed KT role positively -staff engaged in KT activities -greater diffusion of evidence across programs	Moderate (Case report)
Hooge, 2022 [55]	Single group pre-post study	Large academic health system, southeast region, USA, 12-week program; dates not specified	Primary care	11 Advanced practice registered nurses	Virtual mentoring program delivered via Microsoft Teams platform included synchronous training sessions, podcasts, blog and video tutorials, and additional research articles and educational material	Quantitative: -Knowledge and skill for EIDM (Evidence-based Practice Beliefs scale, Evidence-based Practice Implementation scale) -Organizational readiness for EIDM (Organizational Culture and Readiness for System-wide Integration of Evidence-based Practice scale) Qualitative -EIDM facilitators and barriers (open-ended survey)	Compared to baseline, evidence-based practice beliefs scores increased (effect size = 0.71, *p* = 0.018). No significant change in evidence-based practice implementation and organizational culture and readiness for system-wide implementation of evidence-based practice scale scores Barriers to EIDM: -Competing priorities -Time management	High (Quasi-experimental study)
Humphries, 2013 [56]	Case report, no comparator	Regina Qu’Appelle Health Region and Northern Health, Alberta and British Columbia, Canada, 2008–2011	Public health	Management and staff at organizations	The Value Add through Learning and Use of Evidence (VALUE) initiative: -Learning projects (to practice research literacy and skills) -Liaison roles -Research support -Protected time for EIDM activities -Inter-regional collaboration	Findings were described in a narrative case report	Lessons learned included: -Staff turnover was a challenge -Potential benefit to promoting evidence use in staff orientation -Evidence use implementation needs to be directed at multiple levels within the organization -Strategies with ongoing real-time research expertise and support were valued by participants	High (Case report)
Irwin, 2013 [57]	Case report, no comparator	Various healthcare settings, USA, 2009–2010	Primary care	Nursing teams	Institute for Evidence-Based Practice Change program was provided to nurses. This program included a 2.5-day workshop on EIDM, literature searching, and development of an implementation plan, project management, and outcomes measurement. The program also provided an experience mentor for EIDM support for 12-months	Qualitative: -EIDM facilitators and barriers (log entries from the team champion)	Facilitators for EIDM: -Adequate time -Organizational support -Engagement and teamwork -Communication and planning -Maintaining focus on EIDM goals Barriers to EIDM -Competing priorities -Data collection and measurement challenges -Staff turnover	Low (Case report)
Kaplan, 2014 [58]	Case report, no comparator	Magnet-designated hospital, USA, November 1, 2012 to May 10, 2013	Primary healthcare	Nurses across organization	All nurses received an electronic newsletter on EIDM every 2 weeks. A cohort of direct care nurses participated in a series of EIDM workshop to develop, implement, and disseminate an EIDM project	Quantitative: Organizational readiness for integration of EIDM (The Organizational Culture and Readiness for System-Wide Integration of Evidence-Based Practice Scale), EIDM knowledge and skill (Evidence-Based Practice Beliefs Scale), EIDM implementation (The Evidence-Based Practice implementation Scale)	Following the intervention, perceptions of organizational increased. Confidence in implementing EIDM was not associated with EIDM use. Higher education levels was positively associated with nurses’ EIDM use	High (Case report)
Kimber, 2012 [59]	Qualitative	Kinark Child and Family Services, Ontario, Canada, 2006–2010	Child and youth mental health	Staff across organization	Multiple EIDM interventions were implemented, including: -Leadership support -Appointing working group leaders -Dedicated time for EIDM	Qualitative: -EIDM facilitators and barriers (survey)	Facilitators for EIDM -Staff understanding the clinical transformation project and stages -Effective leadership -Change culture inclusive of staff and management, and various disciplines -Cross-program collaboration -Protected time -Evaluation to demonstrate benefits of change Challenges to EIDM: -Underutilization of internal staff -Lack of preparation for change	Moderate (Qualitative)
Mackay, 2019 [60]	Single group pre-post study	Haemodialysis unit of a hospital, Queensland, Australia, 2016–2018	Primary care	All staff at organization	A new nutrition service was established to translate nutrition guidelines into practice to support EIDM through: -Professional development -Evidence-informed recommendations -Multidisciplinary staff involvement -Integrated database prompts	Quantitative: EIDM use, malnutrition prevalence (database audit, Patient-Generated Subjective Global Assessment tool) Qualitative: EIDM facilitators and barriers (clinic observation, team discussion)	There was no significant change in malnutrition categories; most patients (72–80%) began the program well-nourished Facilitators for EIDM: -Establishing processes for best practices -Buy-in from staff and management-in from staff and management -Regular monitoring and feedback Barriers to EIDM: -Limited prior knowledge -Limited time	Moderate (Quasi-experimental study)
Martin-Fernandez, 2021 [61]	Case report, no comparator	Regional health agencies, France, 2017–2019	Public health	Health professionals and decision-makers across regional health agencies	The Transfert de Connaissances en REGion (TC-REG) knowledge translation plan: -Improved access to scientific evidence -EIDM skill development through training, journal clubs and tutoring -Organizational culture development through collaborative workshops, processes, and incentives	Qualitative: -EIDM facilitators and barriers (unstructured interviews) -Use of EIDM (semi-structured interviews)	Facilitators for EIDM: -Understanding of scientific evidence -Confidence in using scientific evidence -Ability to search and find scientific evidence -Motivation to use scientific evidence -Belief that scientific evidence can help to improve practice, develop new frameworks, advocate for their professional activity, and create new partnerships	Moderate (Case report)
Melnyk, 2017 [62]	Single group pre-post study	Washington Hospital Healthcare System, USA, 12 months; dates not specified	Primary care	Service providers, administrators within organizations	EIDM mentors were developed within the healthcare system, through intensive EIDM workshops. Teams of participants implemented and evaluated an EIDM change project within their hospital	Quantitative: Knowledge and skill for EIDM (evidence-based practice beliefs scale, evidence-based practice implementation scale), organizational readiness for EIDM (organizational culture and readiness for system-wide implementation of evidence-based practice scale), patient outcomes (aggregate data from the hospital’s medical records)	Following implementation, -Organizational knowledge and skill for EIDM organization increased (effect size = 0.62; *p* = 0.00) -Organizational implementation of EIDM increased (effect size = 2.3; *p* = 0.00) -Organizational culture and readiness for EBP increased significantly from baseline (*M* = 80.9; *SD* = 90.8) to follow-up (*M* = 90.8; *SD* = 14.7; *t* = 3.9; *p* = 0.00; effect size = 0.70) The following trends were seen in patient outcomes, -Reduction in ventilator days -Decreased pressure ulcer rate -Reduced hospital readmissions for congestive health failure -Increase in patient reported quality of care -Reduced use of formula as a supplement -Decreased wait time for pain medication and decreased length of stay in emergency room	Moderate (Quasi-experimental study)
Miro, 2014 [63]	Single group pre-post study	Fraser Health, Island Health and Vancouver Coastal Health, British Columbia, Canada, 2010—2012	Public health	Organization	Regional health authorities were provided an expert consultant to foster EIDM in land use and transportation plans and policies. The expert worked with staff to develop and facilitate the implementation of the work plans, by conducting a situation assessment, developing and implementing capacity-building plan	Quantitative: Knowledge and skill for land use and transportation plans/policies (survey) Qualitative: Activities completed at the health units (interviews)	Following the intervention, staff reported: -Increased knowledge and skills -Increased awareness of other organizations Facilitators for EIDM -New relationships with colleagues in other health authorities, governments and sectors -Increased opportunities for collaboration -Collaboration between health authorities and local governments -New insights on partnership work Barriers to EIDM -Lack of time and resources -Roles and partnerships not clearly defined -Lack of leadership support and integration across the organization	High (Quasi-experimental study)
Parke, 2015 [64]	Case report, no comparator	Island Health and the University of Alberta, British Columbia, Canada, 2012–2014	Primary care	Whole organization	Scholar-in-residence roles was established to integrate practice, education, and research through collaboration between a health region and a university. Activities included: -Unit-based research teams that conducted literature reviews, literature appraisal -Workshops on writing for publication, research methods skills -Funded research project proposal writing, ethics applications, data collection and analysis -Publications and presentations -Quality improvement through collaboration with community, hospitals and university	Findings were described in a narrative case report	Barriers to EIDM: -Cultural differences between the healthcare and university system -Establishing protected time for research in the health organization -Building relationship between the scholar and hospital staff	Moderate (Case report)
Peirson, 2012 [14]	Qualitative	Peel Public Health, Ontario, Canada, September 2008 to February 2010	Public health	All staff at organization, including leadership	Multiple EIDM interventions were implemented, including: -Hiring new leadership supportive of EIDM -Strategic organizational plan for EIDM -Development of staff knowledge and skills	Qualitative: EIDM facilitators (semi-structured interviews and focus groups, review of documents)	Facilitators for EIDM: -Senior leadership driving EIDM initiatives -Organizational structures (e.g., journal clubs, workshops, library services) -Establishing EIDM specialist roles, training staff in EIDM and encouraging knowledge sharing with co-workers -Supportive organizational culture -Accessible knowledge and sharing knowledge across the organization -Communication around EIDM and its priority to the organization	High (Qualitative)
Plath, 2013 [65]	Qualitative	Non-governmental social service organization, Australia, dates not specified	Social work	Staff across organization	Strategies to promote EIDM were implemented, including: -Leadership commitment to EIDM -Staff champions for EIDM -Establishment of EIDM “communities of practice” teams	Qualitative: -EIDM facilitators and barriers and facilitators (interviews and focus groups)	Facilitators for EIDM: -Dedicated staff roles for research and KT -Supportive leadership -Sufficient time, training and resources for EIDM -Audit and feedback of practices -Building frontline staff skills in EIDM -EIDM “communities of practice” Challenges to EIDM: -Competing priorities -Lack of knowledge and skills -Culture of responding to crises	Moderate (Qualitative)
Roberts, 2020 [66]	Single group pre-post study	Tennessee Department of Health, Tennessee, USA, 2012–2018	Public health	Departments, teams, senior leadership across organization	Volunteers were trained as “Baldrige examiners”, a similar role to knowledge broker. These volunteers supported teams at the local health departments evaluate and improve programming	Quantitative: -Employee satisfaction (survey) -Adoption of new processes (training records) -Integration of new programs (program process reports)	Authors report diffusion of skills across the local health departments. Department staff reported satisfaction with their jobs at rates higher than national averages	Moderate (Quasi-experimental study)
Traynor, 2014 [67]	RCT with control group and case report with no comparator	Public health units, Ontario, Canada, RCT 2003–2007 and case report 2009–2013	Public health	Organization	Two studies implemented Knowledge Brokers who conducted initial and ongoing needs assessments for EIDM, knowledge management and internal network development	Quantitative: social network data, EIDM skills, knowledge and behavior (survey) Qualitative: Knowledge, attitudes and behaviours for EIDM (interviews, journal analysis)	Knowledge brokering intervention was reported to result in increased use of EIDM. Tailoring knowledge broker approaches to the organizational context was most effective. Knowledge brokers were most effective if they were experts in research methodology and public health, as well as being approachable and patient	High (Qualitative)
Van der Zwet, 2020 [68]	Case report, no comparator	Social work Organization, Netherlands, 2013–2015	Social work	Research and development team	Research and development department and long-term collaboration with a university were established to support EIDM	Qualitative: -EIDM facilitators and barriers (semi-structured interviews)	Facilitators for EIDM: -Leadership commitment to research -Qualified staff in EIDM support roles -Research partnerships -Training in EIDM -Targeted recruitment of staff with diverse educational backgrounds Barriers to EIDM: -Negative attitudes towards EIDM -Preference for experiential vs. research knowledge -Culture of crisis-driven practice -Workload, time management, competing priorities	High (Case report)
Ward, 2012 [13]	Case report, no comparator	Peel Public Health, Ontario, Canada, 2010–11 (Year 4 of a 10-year initiative)	Public health	All staff at organization, including leadership	Key elements of the EIDM strategic approach included: -Structured process for research review -Library reference service -Staff development in EIDM knowledge and skills -Dedicated staff time for EIDM -Active engagement with the research community -Accountability for EIDM at all levels of the organization	Findings were described in a narrative case report	After 4 years of implementation, there was systematic and transparent application of research to more than 15 program decisions. EIDM was embedded as a cultural norm within the organization Key lessons identified included: -Identify a senior, influential leader -Commit to a multiyear strategy -Be realistic about the infrastructure needed -Staff support for skill development -Make senior staff accountable for progress -Partner with leading researchers -Invest resources in change management -Measure progress to communicate successes to staff	Moderate (Case report)
Waterman, 2015 [69]	Qualitative	The Greater Manchester Collaboration for Leadership in Applied Health Research and Care, Manchester, United Kingdom; dates not specified	Public health	Organization	KT Associates facilitated the implementation of EIDM. KT Associates joined teams responsible for implementing EIDM along with the clinical lead, academic lead and program manager	Qualitative: -Evaluation of KT Associates’ role and impact (focus group and interviews)	KT Associates contributed to 4 key stages: -Choosing an evidence-based intervention (collecting information, bringing stakeholders together, identify context, build up network) -Planning the evidence-based intervention (collecting evidence, testing the intervention, sharing info, expanding networks, stakeholder meetings) -Co-ordinating and implementing the evidence-based intervention recruit people and build relationships, individualized support, communication, understanding context) -Evaluating evidence-based intervention (data collection/report, patient and staff experiences, celebratory events, poster/presentations)	High (Case report)
Williams, 2020 [70]	Single group pre-post study	Outpatient children’s mental health clinics, Philadelphia, USA, 2013–2017	Primary care	Senior leadership across agencies	Development of organizational leadership and climate for EIDM through training, consultation and technical assistance	Quantitative: -EIDM use (Cognitive-behavioral therapy subscale of the Therapy Procedures Checklist-Family Revised) -Leadership for EIDM (Implementation Leadership Scale) -Organizations’ climates for EIDM (Implementation Climate Scale) -Perceptions of leader’s transformational leadership (Multifactor Leadership Questionnaire) -Attitudes toward EIDM (Evidence-based Practice Attitudes Scale)	Organizational climates supportive of EIDM were associated with: -Strong leadership for EIDM (d = 0.92, *p* = 0.017) -Increased use of EIDM (d = 0.55, *p* = 0.007) There was no association between clinicians’ attitudes towards EIDM and their use of EIDM	High (Quasi-experimental study)
Williams, 2019 [71]	Single group pre-post study	Metabolic specialist centres, Australia and New Zealand, 2015–2017	Primary care	Metabolic dietetic service within organization	The metabolic dietetic service established: -Electronic referral alert -Metabolic sick day nutrition plans available to all clinical staff -Metabolic diet codes and specialised formula recipes	Quantitative: Admissions for patients with inborn errors of metabolism (chart audit)	There was a reduction in total admissions of patients with inborn errors of metabolism (36 vs. 11 across the audit periods; unclear if this was a statistically significant finding.)	Moderate (Quasi-experimental study)
Williams, 2017 [72]	Single group pre-post study	Children’s mental health agencies, large midwestern urban area, USA, 2010–2013	Primary care	CEOs and administrators, and front-line clinical teams at organizations	External facilitators supported leadership, staff and an internal liaison. Principles of EIDM were integrated into the organizations’ operating procedures. Organizational infrastructure and tools to enable EIDM were developed. Staff and leadership mental models to support EIDM were enabled	Quantitative: Intentions to adopt EIDM, barriers to EIDM (surveys), Unit-level enactment of Availability, Responsiveness, and Continuity principles and completion of planned activities (ARC principles questionnaire), Organizational proficiency culture for EIDM (Organizational Social Context measure)	Following implementation, clinicians exhibited: -Higher odds of adopting EIDM (OR = 3.19, *p* = 0.003) -Greater use of EIDM with clients (*p* = 0.003) -Fewer EIDM barriers (*p* = 0.026) Intention to use EIDM was the only predictor of EIDM adoption (*p* = 0.032) and EIDM use (*p* = 0.002)	High (Quasi-experimental study)

Study designs included case reports (*n* = 18), single group pre-/post-test studies (*n* = 10), qualitative studies (*n* = 7), and randomized controlled trials (RCTs) (*n* = 2). Both RCTs evaluated the implementation of organizational EIDM.

Studies reported quantitative (*n* = 11), qualitative (*n* = 20), or both quantitative and qualitative results (*n* = 6). For the studies that reported quantitative results, measures included EIDM implementation, EIDM-related beliefs and behaviours, organizational priorities for EIDM, and patient care quality indicators. Quantitative measures were heterogenous and did not allow meta-analysis. Qualitative findings were generated through formal qualitative analysis (*n* = 19) or descriptive case reports (*n* = 7). Most qualitative results included facilitators and barriers to implementation (*n* = 16).

### Study quality

The critical appraisal checklist used to assess each study is indicated in Table 1. Single group, pre-/post-test studies were evaluated according to the JBI Checklist for Quasi-experimental Studies [35].

A lack of control groups contributed to the risk of bias. Most included studies were rated Moderate or High quality according to their respective quality assessment tools. Full quality assessments for each article are included in Appendix 2. Therefore, the overall methodological quality for this body of literature was rated as Moderate.

### Strategies for implementing organization-wide EIDM

Due to the heterogeneity of study designs, interventions, and outcomes, it was not possible to determine which EIDM implementation strategies are more effective compared to others. Implementation strategies included the establishment of Knowledge Broker-type roles, building the EIDM capacity of staff, and research or academic partnerships. These strategies are listed in Table 2.

**Table 2:** Strategies for implementation of organization-wide EIDM

**Strategy**	**Studies**
Establishing specialized roles, e.g., Knowledge Brokers	[20, 41, 44, 47, 48, 51, 52, 54–57, 59, 60, 62–67, 69, 71, 72]
Building staff capacities for EIDM through edutation and training	[13, 14, 39, 40, 42, 43, 46, 49, 50, 58, 61]
Research or academic partnerships	[45, 53, 68]

Evaluation of strategies implemented by studies in this review was often qualitative and described facilitators and barriers, rather than quantitatively measuring effectiveness. However, it is possible to explore EIDM implementation strategies and factors that appear to contribute to or inhibit success. The most common strategy implemented in included studies was the establishment of Knowledge Broker-type roles [20, 41, 44, 47, 48, 51, 52, 54–57, 59, 60, 62–67, 69, 71, 72]. Studies described roles differently (e.g., “Evidence-based Practice Facilitator”, “Evidence Facilitator”, “EIDM Mentor”). These roles all served to support EIDM across organizations through knowledge sharing, evidence synthesis, implementation, and other EIDM-related activities. In some studies, new staff were hired to Knowledge Broker roles, or developed among existing staff, while in others, Knowledge Brokers were contracted from external organizations. Knowledge Broker strategies were mostly implemented in parallel with other EIDM implementation strategies, such as capacity building for staff, integrating EIDM into decision-making processes and development of leadership to support EIDM. When these strategies were evaluated quantitatively for organizational capacity, culture and implementation of EIDM, most studies found positive results, such as increased scores for organizational climates supporting EIDM, improved attitudes toward EIDM, or the integration of EIDM into processes [44, 52, 54, 62, 66, 67, 71, 72], although some studies found no change [55, 60] following implementation of Knowledge Broker roles. Qualitatively, most studies described facilitators and barriers to EIDM, either through formal qualitative analysis or case report [14, 20, 39–43, 45, 47, 48, 52, 55, 57, 59–61, 64, 65, 68]. Facilitators included organizational culture with supportive leadership and staff buy-in, expectations to use evidence to inform decisions, accessible knowledge, and integration of EIDM into processes and templates. Barriers included limited time and competing priorities, staff turnover, and lack of understanding and support from management.

Ten included studies focused primarily on building EIDM capacity of existing staff at the organization, often at multiple levels (e.g., front-line service providers, managers, and leadership) [13, 14, 39, 40, 42, 43, 46, 49, 50, 58, 61]. Capacity building was typically done through EIDM-focused workshops, often with ongoing follow up support from workshop facilitators. While studies often measured changes in individual knowledge and skill for EIDM for workshop participants, organizational change for EIDM was reported qualitatively, either through formal qualitative analysis or through a case report. Facilitators for EIDM in these ten studies included organizational culture with supportive leadership and staff buy-in, dedicated staff roles to support EIDM, opportunities to meet and discuss EIDM (e.g., communities of practice, journal clubs), knowledge sharing across the organization, expectations to use evidence to inform decisions, accessible knowledge, and integration of EIDM into processes and templates. Barriers included limited time and competing priorities, staff turnover, and negative attitudes toward EIDM.

Research or academic partnerships and networks were the main strategy described in three case reports [45, 53, 68]. These involved establishing collaborations, either through universities or non-governmental health organizations, that provided direct EIDM support. These strategies were not evaluated quantitatively but described facilitators and barriers to effective cross-sector collaborations. Facilitators for EIDM included supportive leadership and management, dedicated staff roles to support EIDM, EIDM knowledge and skill development for staff, and regular communication between partners. Barriers included limited time and competing priorities, preference for experiential over research evidence, and negative attitudes toward EIDM.

Overall, studies described successes in implementing EIDM across organizations, citing several common key facilitators and barriers. To instigate behaviour change, strategies must address capability for change, which may be achieved by building staff capacity, establishing dedicated support roles, improving access to evidence, and sharing knowledge across the organization. Strategies must also enable opportunities for change, which may be supported through forums for EIDM learning and practice, protecting time for EIDM, integrating EIDM into new or existing roles, and adding EIDM to processes and templates. Behaviour change also requires motivation, which may be built through a supportive organizational culture, expectations to use EIDM, recognition and positive reinforcement, and strong support from leadership.

### Key considerations for implementing EIDM

**Fig. 2:**
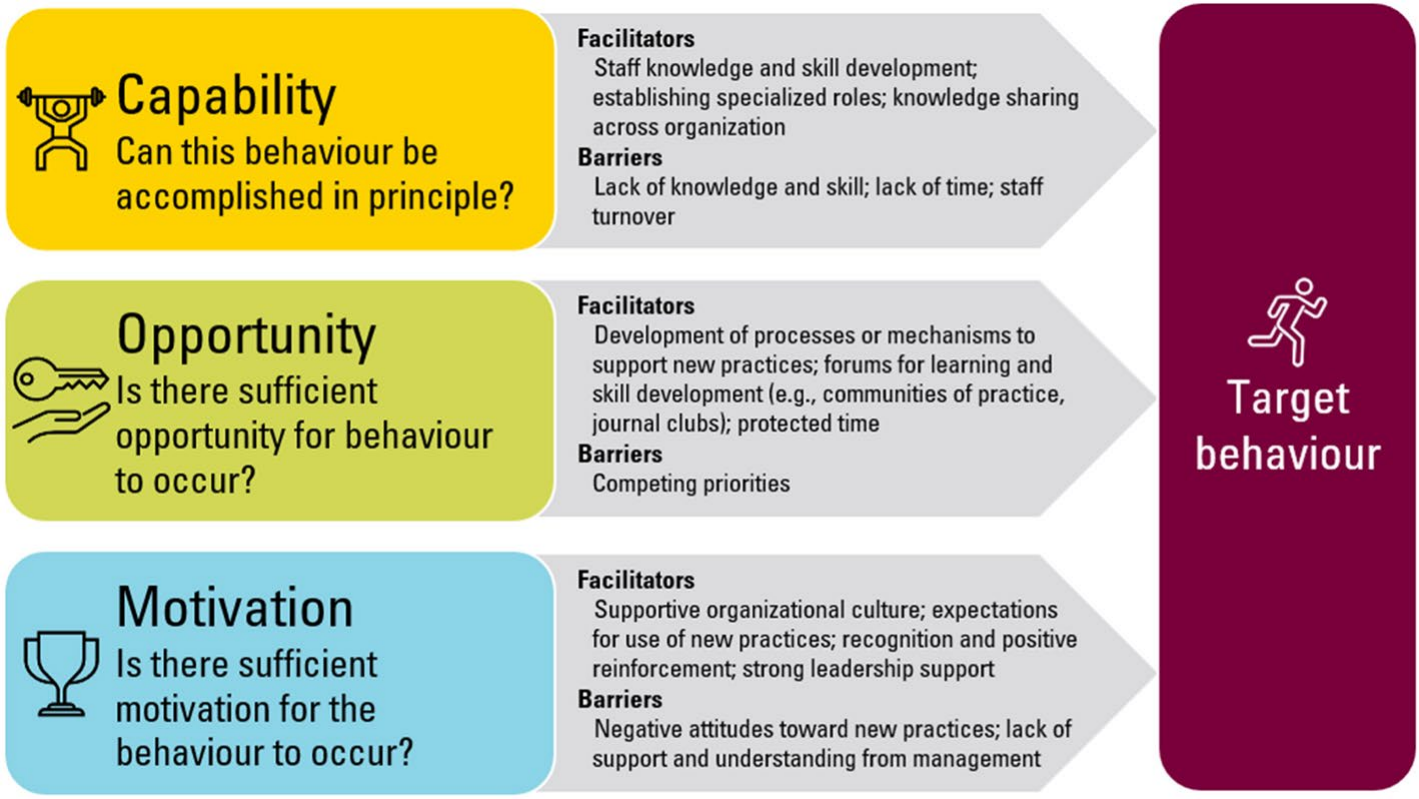
COM-B Model for behaviour change with facilitators and barriers for implementation of organization-wide EIDM

Many of the facilitators and barriers to EIDM are common across strategies explored by the studies included in this review. To conceptualize these factors, they were mapped to the COM-B model for behaviour change [21] in Fig. 2.

Within the capability component of the COM-B model, staff knowledge and skill development were included as a facilitator. Studies included in this review demonstrated that knowledge and skill for EIDM supported the use of evidence in decision making [13, 14, 39, 40, 42, 43, 46, 49, 50, 58, 61]. The establishment of specialized or dedicated roles for EIDM, such as Knowledge Broker roles, was included in the capability component of the COM-B model, since Knowledge Broker roles support the capacity of organizations and their staff to use evidence-informed approaches [20, 41, 44, 47, 48, 51, 52, 54–57, 59, 60, 62–67, 69, 71, 72]. Finally, knowledge sharing across organizations was described as a facilitator for EIDM by several of the studies that built staff capacity for EIDM or established Knowledge Broker roles [13, 48, 49, 51, 52, 54, 56, 59, 61, 65]. Barriers to the capability for EIDM behaviours include staff turnover and subsequent knowledge loss [14, 20, 56]. Staff turnover is especially challenging for interventions that involve staff in dedicated Knowledge Broker roles and interventions that build the knowledge and skill for staff to engage in evidence use [14, 20, 56]. In some cases, individuals who are trained in the Knowledge Broker role are then promoted to new roles or management and have fewer opportunities to apply their Knowledge Broker skills [20].

The opportunity portion of the COM-B model reflects whether there is opportunity for new behaviour to occur. The development of processes and mechanisms that support new practices can act as a reminder for staff, and may include re-design of planning or decision-making templates to capture supporting evidence, or adding EIDM-related items to agendas for regular meetings [41, 47, 53, 60]. Forums for learning and skill development provide staff with opportunities to gain knowledge and practice newly acquired skills in group settings, such as communities of practice or journal clubs [48, 56, 61, 65]. Finally, protected time to apply EIDM was found to be a facilitator for opportunity in the COM-B model [20, 47, 57, 59, 65], while competing priorities were found to be a barrier [20, 39, 40, 52, 55, 57, 60, 64, 65].

The final influencer in the COM-B model, motivation, reflects whether there is sufficient motivation for a new behaviour to occur. Facilitators include supportive organizational culture [14, 20, 43, 47, 57, 59], expectations for new practices to occur [20, 40], recognition and positive reinforcement [52, 59, 60, 65], and strong leadership support [14, 20, 39, 40, 43, 47, 56, 59, 65, 68]. Barriers to motivation included a lack of understanding or support from management [20], and negative attitudes toward change [20, 52, 59, 68].

## Discussion

Strategies to implement EIDM across organizations include establishing specialized roles, providing staff education and training, developing processes or mechanisms to support new practices, and demonstrating leadership support. Facilitators and barriers for these strategies align with the COM-B model for behaviour change, which outlines capability, opportunity, and motivation as influencers of behaviour (Fig. 2). The COM-B model provides a comprehensive framework for the factors that influence behaviour change and has provided a valuable structure for examining barriers and facilitators to behaviour change in public health and related fields [73–76].

The capability section of the COM-B model reflects whether the intended audience possess the knowledge and skill for a new behaviour. Findings from this review establish facilitators for EIDM implementation capability, including the development of staff knowledge and skill, establishing specialized roles, and knowledge sharing across the organization. The development of staff knowledge and skill for EIDM are a necessary component to ensure EIDM in practice, however, literature has found that the organization-wide impact of conducting only individual-level knowledge and skill development is limited [77–79]. While knowledge and skill development are a necessary component to EIDM practice, they must be supported by other components to have an impact beyond the individual. Other strategies that support the use of newly gained knowledge and skills include the establishment of specialized roles for EIDM. Another strategy to support the use of EIDM is the establishment of dedicated staff roles, such as Knowledge Brokers. Knowledge Broker roles have been used across diverse contexts and show promise in supporting organization-wide EIDM implementation [20, 22, 23, 67, 80–83]. One facilitator for Knowledge Broker roles was knowledge sharing across the organization. Factors that influence the success of staff in Knowledge Broker roles align with those mapped to opportunity and motivation in the COM-B model, including the integration of EIDM into processes, knowledge sharing, and supportive organizational culture [20, 22, 47, 67, 84, 85]. Knowledge Brokers can also help facilitate knowledge sharing across the organization, which was another facilitator mapped to the capability level of the model [20, 47, 84, 85]. Knowledge sharing refers to the shared learning, knowledge products and resources for EIDM. At large public health organizations, it can be challenging to facilitate knowledge sharing between teams and departments [86, 87]. Integrating technology can help; there have been some advances driven by the COVID-19 pandemic, such as the development of knowledge sharing platforms [88–91]. Public health organizations seeking to implement EIDM should invest in their knowledge sharing infrastructure.

At the capability level of the COM-B model, staff turnover was a barrier to EIDM implementation. Organizations that implement these strategies should be cognizant of the potential for knowledge loss due to staff turnover when selecting staff for Knowledge Broker roles or capacity building opportunities.

Facilitators for organizational EIDM opportunity include the development of processes or mechanisms to support new practices, forums for learning and skill development, and protected time. The use of reminders for organizational behaviour change and implementation of clinical practice guidelines has been shown to be an effective strategy across many contexts [92–95]. Organizations seeking to implement EIDM should consider revising current templates and processes to support their initiatives. Another facilitator included forums for shared learning and skill development. Other literature shows that these forums can be effective in developing knowledge and skill and should foster an environment of learning without fear of reprisal [96, 97]. Finally, protected time for EIDM was a facilitator and competing priorities were a barrier. In public health practice, staff are often challenged with high workloads, so that EIDM may be viewed as an additional burden rather than a means to improve practice [98, 99]. For an EIDM approach to be practiced, staff must be provided with sufficient time to apply and practice skills. Organizations should consider involving middle management who oversee staff time allocations, rather than only senior leadership, to help ensure that staff are provided with the time they need and that expectations are adjusted accordingly [20, 23].

At the motivation level of the COM-B model, supportive organizational culture was mapped as a facilitator. The influence of organizational culture on evidence-informed practice at health organizations has been explored in a previous systematic review by Li et al. [100]. This systematic review of organizational contextual factors that influence evidence-based practice included 37 studies conducted in healthcare-related settings. Findings align with facilitators identified above, especially leadership support, which was found to impact evidence-based practice as well as all other factors that influence evidence-based practice [100]. The review also found that monitoring and feedback contributed to implementation of evidence-based practice, which aligns with recognition and positive reinforcement in the COM-B model above [100]. Notably, another factor that was mapped to the COM-B model was the expectation for new practices to occur, which was not explicitly identified as an influence on practice [100]. While Li et al. acknowledge that leadership that neglects to hold staff accountable are detrimental to implementation of EIDM, this accountability and clear expectations for change practice were a stronger finding in this current rapid systematic review.

The need for leadership support aligns with opportunity, since it is often management that determines the allocation of staff time for EIDM [20, 23]. Attitudes and the belief that EIDM is associated with positive outcomes is a key factor in overall competence for EIDM [101]. Efforts to address negative attitudes within staff, especially at the leadership level, may improve implementation of EIDM.

While this review provides a comprehensive overview of interventions to support EIDM in public health and related organizations, it does have some limitations. Given the heterogeneity of included studies, it was not possible to discern which implementation strategies for EIDM are more effective compared to others. Knowledge Broker roles, building capacity for EIDM, and research-academic partnerships were all shown to contribute to EIDM, but study findings do not support one strategy as superior to others. Given the highly contextual nature of these interventions, it is likely that the relative effectiveness of different interventions depends on the organization’s unique set of characteristics. Evaluation is also critical to determine if change efforts are successful or need to be adjusted. It is possible that a combination of strategies would maximize the likelihood that diverse needs of staff are met. Rigorous studies to evaluate this hypothesis are needed.

Most studies included in this review are non-randomized studies of interventions. Given the importance of context in organizational change, randomized controlled trial designs may not be well-suited to evaluate studies of EIDM implementation [102]. High-quality single-group studies, such as prospective cohort analytic studies evaluated with validated measures or qualitative descriptive analyses of case studies with thorough descriptions of interventions and context, may be more appropriate designs for designing future initiatives in this field. However, arguments have been made for the use of randomized trial designs in implementation research [103]. Foy et al. advocate for overcoming contextual barriers by using innovative trial designs, such as the multiphase optimization strategy approach, where a series of trials identify the most promising single or combined intervention components, or the sequential multiple assignment randomized trial approach, where early results inform tailoring of adaptive interventions [103]. These designs may be a promising approach to conducting trials within highly contextual settings. Another viewpoint is that perhaps it may not be essential to determine if one strategy is superior to another, but rather that strategies build a larger, multi-strategy approach to implementation [104]. There may be greater benefit to determining the conditions under which various strategies are effective [104].

A limitation in this review’s methodology is that the review was completed following a rapid review protocol to ensure timely completion. Modifications of a systematic review approach included the use of a single reviewer for screening and using an unblinded reviewer to check quality assessment and data extraction. This may have contributed to some bias within the review, due to the reviewers’ interpretations of studies. To minimize this bias, there were efforts to calibrate screening, quality assessment and data extraction using a subset of studies.

This review provides a synthesis of strategies for the organization-wide implementation of EIDM, and an in-depth analysis of their facilitators and barriers in public health organizations. Facilitators and barriers mapped to the COM-B model for behaviour change can be used by organizational leadership to drive organizational change toward EIDM.

## Conclusion

This rapid systematic review explored the implementation of EIDM at the organizational level of public health and related organizations. Despite the similarity of these implementation challenges, studies used distinct strategies for implementation, including the establishment of dedicated roles to support EIDM, building staff capacities, research or academic partnerships, and integrating evidence into processes or mechanisms. Facilitators and barriers mapped to the COM-B model provide key guidance for driving organizational change to evidence-informed approaches for all decisions.

### Supplementary Information

**Supplementary Material 1.**

**Supplementary Material 2.**

## Data Availability

All data generated or analysed during this study are included in this published article and its supplementary information files.

## Abbreviations

EIDM: Evidence-informed Decision Making
EBP: Evidence-based Practice
EIP: Evidence-informed Practice
GRADE: Grading of Recommendations, Assessment, Development and Evaluations
JBI: Joanna Briggs Institute
KT: Knowledge Translation
RCT: Randomized Controlled Trial
