# Supplementary material for: Strategies to implement evidence-informed decision making at the organizational level: a rapid systematic review
Source: BMC Health Serv Res. 2024 Apr 1;24:405. doi: 10.1186/s12913-024-10841-3 (PMC10983660; doi:10.1186/s12913-024-10841-3)
Supplement: Supplementary file 1 — Supplementary Material 1. [file 12913_2024_10841_MOESM1_ESM.docx]

**Appendix 1: Search Strategy**

On March 18, 2022, the following databases were searched using the search terms listed.

## Medline

| **#** | **Query** |
| --- | --- |
| 1 | implement*.mp. |
| 2 | integrat*.mp. |
| 3 | "knowledge broker*".mp. |
| 4 | transform*.mp. |
| 5 | or/1-4 |
| 6 | Organizational Culture/ |
| 7 | Organizational Innovation/ |
| 8 | organizational case studies/ |
| 9 | or/6-8 |
| 10 | 5 and 9 |
| 11 | Change Management/ |
| 12 | Capacity Building/ |
| 13 | or/11-12 |
| 14 | 10 or 13 |
| 15 | "EIDM".mp. |
| 16 | "EBP".mp. |
| 17 | "EBM".mp. |
| 18 | ("evidence-based" or "evidence based").mp. |
| 19 | ("evidence-informed" or "evidence informed").mp. [mp=title, abstract, original title, name of substance word, subject heading word, floating sub-heading word, keyword heading word, organism supplementary concept word, protocol supplementary concept word, rare disease supplementary concept word, unique identifier, synonyms] |
| 20 | "knowledge translation".mp. |
| 21 | "KT".mp. |
| 22 | "KE".mp. |
| 23 | "knowledge exchange".mp. |
| 24 | Evidence-Based Practice/ |
| 25 | Evidence-Based Medicine/ |
| 26 | Evidence-Based Nursing/ |
| 27 | Evidence-Based Dentistry/ |
| 28 | or/15-27 |
| 29 | 14 and 28 |

## Embase

| **#** | **Query** |
| --- | --- |
| 1 | implement*.mp. |
| 2 | integrat*.mp. |
| 3 | "knowledge broker*".mp. |
| 4 | transform*.mp. |
| 5 | or/1-4 |
| 6 | organizational culture/ |
| 7 | organizational innovation.mp. |
| 8 | or/6-7 |
| 9 | 5 and 8 |
| 10 | change management/ |
| 11 | capacity building/ |
| 12 | or/10-11 |
| 13 | 9 or 12 |
| 14 | "EIDM".mp. |
| 15 | "EBP".mp. |
| 16 | "EBM".mp. |
| 17 | ("evidence-based" or "evidence based").mp. |
| 18 | ("evidence-informed" or "evidence informed").mp. |
| 19 | "knowledge translation".mp. |
| 20 | "KT".mp. |
| 21 | "KE".mp. |
| 22 | "knowledge exchange".mp. |
| 23 | evidence based practice/ |
| 24 | evidence based practice center/ |
| 25 | evidence based nursing/ |
| 26 | evidence based medicine/ |
| 27 | evidence based dentistry/ |
| 28 | or/14-27 |
| 29 | 13 and 28 |

## Ovid Emcare

| **#** | **Query** |
| --- | --- |
| 1 | implement*.mp. |
| 2 | integrat*.mp. |
| 3 | "knowledge broker*".mp. |
| 4 | transform*.mp. |
| 5 | or/1-4 |
| 6 | organizational culture/ |
| 7 | organizational innovation.mp. |
| 8 | or/6-7 |
| 9 | 5 and 8 |
| 10 | change management/ |
| 11 | capacity building/ |
| 12 | or/10-11 |
| 13 | 9 or 12 |
| 14 | "EIDM".mp. |
| 15 | "EBP".mp. |
| 16 | "EBM".mp. |
| 17 | ("evidence-based" or "evidence based").mp. |
| 18 | ("evidence-informed" or "evidence informed").mp. |
| 19 | "knowledge translation".mp. |
| 20 | "KT".mp. |
| 21 | "KE".mp. |
| 22 | "knowledge exchange".mp. |
| 23 | evidence based practice/ |
| 24 | evidence based medicine/ |
| 25 | evidence based nursing/ |
| 26 | evidence based dentistry/ |
| 27 | or/14-26 |
| 28 | 13 and 27 |

## Global Health Database

| **#** | **Query** |
| --- | --- |
| 1 | implement*.mp. [mp=abstract, title, original title, broad terms, heading words, identifiers, cabicodes] |
| 2 | integrat*.mp. [mp=abstract, title, original title, broad terms, heading words, identifiers, cabicodes] |
| 3 | "knowledge broker*".mp. |
| 4 | transform*.mp. |
| 5 | or/1-4 |
| 6 | organizational case studies.mp. |
| 7 | ("organizational culture" or "organisational culture").mp. [mp=abstract, title, original title, broad terms, heading words, identifiers, cabicodes] |
| 8 | ("organizational case studies" or "organisational case studies").mp. [mp=abstract, title, original title, broad terms, heading words, identifiers, cabicodes] |
| 9 | or/6-8 |
| 10 | 5 and 9 |
| 11 | capacity building.mp. |
| 12 | change management.mp. |
| 13 | or/11-12 |
| 14 | 10 or 13 |
| 15 | "EIDM".mp. |
| 16 | "EBP".mp. |
| 17 | "EBM".mp. |
| 18 | ("evidence-based" or "evidence based").mp. |
| 19 | ("evidence-informed" or "evidence informed").mp. |
| 20 | "knowledge translation".mp. |
| 21 | "KT".mp. |
| 22 | "KE".mp. |
| 23 | "knowledge exchange".mp. |
| 24 | or/15-23 |
| 25 | 14 and 24 |

PsycInfo

| **#** | **Query** |
| --- | --- |
| 1 | implement*.mp. |
| 2 | integrat*.mp. |
| 3 | "knowledge broker*".mp. |
| 4 | transform*.mp. |
| 5 | or/1-4 |
| 6 | Organizational climate/ |
| 7 | Organizational Behavior/ |
| 8 | or/6-7 |
| 9 | 5 and 8 |
| 10 | Organizational Change/ |
| 11 | 9 or 10 |
| 12 | "EIDM".mp. |
| 13 | "EBP".mp. |
| 14 | "EBM".mp. |
| 15 | ("evidence-based" or "evidence based").mp. |
| 16 | ("evidence-informed" or "evidence informed").mp. |
| 17 | "knowledge translation".mp. |
| 18 | Knowledge Transfer/ |
| 19 | "KT".mp. |
| 20 | "KE".mp. |
| 21 | "knowledge exchange".mp. |
| 22 | exp Evidence Based Practice/ |
| 23 | or/12-22 |
| 24 | 11 and 23 |

## Web of Science

## (((implement* OR integrat* OR "knowledge broker*" OR transform*) AND (“Organizational Culture” OR “Organizational Innovation” OR “organizational case studies”)) OR (“Change Management” OR “Capacity Building”)) AND ("EIDM" OR "EBP" OR "EBM" OR ("evidence-based" or "evidence based") OR ("evidence-informed" or "evidence informed") "knowledge translation" OR "KT" OR "KE" OR "knowledge exchange")

In addition to the above searches, literature by key contributors was screened through a targeted search of publications.

Leena Augimeri

Melanie Barwick

Peter Pronovost

Byron J Powell

Shelley Wilkinson

Kate Davis

Megan Corlis

Tabatha Rando
