# Supplementary material for: Strategies to implement evidence-informed decision making at the organizational level: a rapid systematic review
Source: BMC Health Serv Res. 2024 Apr 1;24:405. doi: 10.1186/s12913-024-10841-3 (PMC10983660; doi:10.1186/s12913-024-10841-3)
Supplement: Supplementary file 2 — Supplementary Material 2. [file 12913_2024_10841_MOESM2_ESM.docx]

**Appendix 2: Quality Assessments of Included Studies**

**Table A1a: Studies assessed using JBI Checklist for RCTs**

| Study | JBI Checklist for Randomized Controlled Trials | | | | | | | | | | | | | | |
| --- | --- | --- | --- | --- | --- | --- | --- | --- | --- | --- | --- | --- | --- | --- | --- |
|  | 1. True randomization | 2. Allocation concealment | 3. Similar groups at baseline | 4. Participants blinded | 5. Those delivering treatment blinded | 6. Outcome assessors blinded | 7. Identical treatment of groups | 8. Follow up complete | 9. Participants analyzed in groups | 10. Outcomes measured in same way | 11. Reliable outcome measurement | 12. Appropriate statistical analysis | 13. Trial design appropriate | Overall score* | Rating^†^ |
| Brownson, 2017 (69) | No | No | Yes | No | No | No | Yes | No | No | Yes | Yes | Yes | Yes | 6 | Moderate |
| Flaherty, 2021 (68) | Yes | Yes | Yes | No | No | No | Yes | No | Yes | Yes | Yes | Yes | Yes | 9 | Moderate |

*Only items that received a “Yes” were counted toward the overall score.

^†^Ratings were based on the total criteria met by the study (1-4 Low; 5-9 Moderate, 10-13 High).

**Table A1b: Studies assessed using JBI Checklist for Quasi-Experimental Studies**

| Study | JBI Checklist for Quasi-Experimental Studies | | | | | | | | | | |
| --- | --- | --- | --- | --- | --- | --- | --- | --- | --- | --- | --- |
|  | 1. Clear cause and effect | 2. Similar participants in comparisons | 3. Participants received similar treatment | 4. Control group | 5. Multiple outcome measurements | 6. Follow up complete or differences described | 7. Outcomes measured in same way | 8. Reliable outcome measurement | 9. Appropriate statistical analysis | Overall score* | Rating^†^ |
| Breckenridge-Sproat, 2015 (53) | Yes | No | No | No | No | Yes | No | Yes | Yes | 4 | Moderate |
| Dobbins, 2019 (49) | Yes | Yes | Yes | No | Yes | Yes | Yes | No | No | 6 | Moderate |
| Hooge, 2022 (45) | Yes | Yes | Yes | No | Yes | No | Yes | Yes | Yes | 7 | High |
| Mackay, 2019 (50) | Yes | Yes | Yes | No | Yes | No | No | Yes | Yes | 6 | Moderate |
| Melnyk, 2017 (52) | Yes | No | No | No | No | Yes | No | Yes | Yes | 4 | Moderate |
| Miro, 2014 (47) | Yes | Yes | Yes | No | Yes | Yes | Yes | Yes | No | 7 | High |
| Roberts, 2020 (48) | Yes | No | No | No | No | No | Yes | Yes | Yes | 4 | Moderate |
| Williams, 2019 (51) | Yes | Yes | No | No | Yes | No | No | Yes | Yes | 5 | Moderate |
| Williams, 2017 (46) | Yes | Yes | Yes | Yes | Yes | No | Yes | Yes | Yes | 8 | Low |

*Only items that received a “Yes” were counted toward the overall score.

^†^Ratings were based on the total criteria met by the study (1-3 Low; 4-6 Moderate, 7-9 High).

**Table A1c: Studies assessed using JBI Checklist for Qualitative Research**

| Study |  | JBI Checklist for Qualitative Research | | | | | | | | | | | |
| --- | --- | --- | --- | --- | --- | --- | --- | --- | --- | --- | --- | --- | --- |
|  | 1. Congruity with research methods | | 2. Congruity with research question | 3. Congruity with data collection methods | 4. Congruity with data analysis | 5. Congruity with results interpretation | 6. Statement locating the researcher theoretically | 7. Researcher influence addressed | 8. Participants adequately represented | 9. Ethical approval | 10. Conclusions flow from data | Overall score* | Rating^†^ |
| Allen, 2018 (61) | Yes | | Yes | Yes | Yes | Mp | No | No | Yes | Yes | Yes | 7 | Moderate |
| Clark, 2022 (20) | Yes | | Yes | Yes | Yes | Yes | No | No | Yes | Yes | Yes | 8 | High |
| Gifford, 2014 (55) | Yes | | Yes | Yes | Yes | Yes | No | No | Yes | Yes | Yes | 8 | High |
| Kimber, 2012 (58) | No | | Yes | Yes | Yes | Yes | No | No | Yes | Yes | Yes | 7 | Moderate |
| Peirson, 2012 (14) | No | | Yes | Yes | Yes | Yes | Yes | No | Yes | Yes | Yes | 8 | High |
| Plath, 2013 (57) | No | | Yes | Yes | No | Yes | No | No | Yes | Yes | No | 5 | Moderate |
| Traynor, 2014 (56) | Yes | | Yes | Yes | Yes | Yes | No | No | Yes | Yes | Yes | 8 | High |
| Waterman, 2015 (54) | Yes | | Yes | Yes | Yes | Yes | No | Yes | Yes | Yes | Yes | 9 | High |

*Only items that received a “Yes” were counted toward the overall score.

^†^Ratings were based on the total criteria met by the study (1-4 Low; 5-7 Moderate, 8-10High).

**Table A1d: Studies assessed using JBI Checklist for Case Reports**

| Study | JBI Checklist for Case Reports | | | | | | | | | |
| --- | --- | --- | --- | --- | --- | --- | --- | --- | --- | --- |
|  | 1. Demographics described | 2. Presented as a timeline | 3. Current condition described | 4. Assessment described | 5. Intervention described | 6. Post-intervention condition described | 7. Adverse events described | 8. Takeaway lessons | Overall score* | Rating^†^ |
| Allen, 2018 (62) | Yes | Yes | No | Yes | Yes | No | No | No | 4 | Moderate |
| Augustino, 2020 (38) | Yes | Yes | No | No | Yes | Yes | No | Yes | 5 | Moderate |
| Awan, 2015 (65) | No | No | No | Yes | Yes | Yes | No | Yes | 4 | Moderate |
| Bennett, 2016 (64) | Yes | Yes | No | Yes | Yes | No | Yes | Yes | 7 | High |
| Brodowski, 2018 (63) | Yes | Yes | No | No | Yes | Yes | No | Yes | 5 | Moderate |
| Elliott, 2021 (42) | Yes | Yes | Yes | No | Yes | No | Yes | Yes | 6 | Moderate |
| Fernandez, 2014 (66) | Yes | Yes | Yes | Yes | Yes | Yes | No | Yes | 7 | High |
| Gallagher-Ford, 2014 (40) | No | No | No | No | No | Yes | No | Yes | 2 | Low |
| Haynes, 2020 (70) | No | No | No | Yes | Yes | Yes | No | Yes | 4 | Moderate |
| Hitch, 2019 (43) | Yes | No | No | Yes | Yes | No | Yes | Yes | 5 | Moderate |
| Humphries, 2013 (39) | Yes | No | No | No | Yes | Yes | No | Yes | 4 | Moderate |
| Irwin, 2013 (41) | No | No | No | Yes | No | No | No | Yes | 2 | Low |
| Kaplan, 2014 (67) | Yes | No | No | Yes | Yes | Yes | No | No | 4 | Moderate |
| Martin-Fernandez, 2021 (59) | Yes | No | No | Yes | Yes | No | No | Yes | 4 | Moderate |
| Parke, 2015 (44) | Yes | Yes | No | No | Yes | No | No | Yes | 4 | Moderate |
| Van der Zwet, 2020 (60) | Yes | Yes | No | Yes | Yes | Yes | Yes | Yes | 8 | High |
| Ward, 2012 (13) | Yes | No | No | Yes | Yes | Yes | No | Yes | 5 | Moderate |

*Only items that received a “Yes” were counted toward the overall score.

^†^Ratings were based on the total criteria met by the study (1-3 Low; 4-6 Moderate, 7-8 High).
